# ED‐to‐community care transitions for persons living with dementia

**DOI:** 10.1002/alz.091845

**Published:** 2025-01-09

**Authors:** Cameron Gettel

**Affiliations:** ^1^ Yale School of Medicine, New Haven, CT USA

## Abstract

**Objectives:**

Little data exists to date regarding effective interventions to improve emergency department (ED)‐to‐community care transitions for persons living with cognitive impairment (PLWCI) and their care partners. We sought to develop, refine, and pilot test the innovative pairing of artificial intelligence (AI)‐enabled digital advisors and an occupational therapist‐led care coach intervention to improve ED‐to‐community care transitions for PLWCI and their care partners.

**Methods:**

We used a mixed methods, multi‐phased approach to develop the intervention, with PLWCI and care partners sampled from the LiveWell Dementia Specialists network. In the first phase, we conducted semi‐structured focus groups and two researchers coded the professionally transcribed data using a combined deductive and inductive approach. In the second phase, we performed a 2‐day design thinking workshop using a user‐centered design approach, involving PLWCI, care partners, health care professionals, community‐based partner organizations, technologists, and AI experts. In the final phase, we pilot tested the paired intervention among PLWCI and care partners experiencing ED‐to‐community care transitions, assessing: 1) intervention outcomes and 2) patient‐ and care partner‐centered outcomes.

**Results:**

In the first phase, we performed two focus groups, involving a total of 16 participants, with PLWCI and care partners expressing: 1) concerns that their unique needs related to cognition were not addressed in the ED setting, 2) frustration with the ED discharge process and their perceived ability to follow‐up in the outpatient setting and obtain desired resources, and 3) openness to the incorporation of AI to facilitate successful ED‐to‐community care transitions. In the second phase, 23 individuals participated in the design thinking workshop. We characterized perceived barriers and facilitators for intervention adoption during open forum discussions and identified 10 Lessons Learned for future investigators to consider in intervention development. In the third phase, 16 participants have completed all surveys up to 30‐day follow‐up with evidence of improvements in the 4‐item Zarit Caregiver Burden scale (13.9 to 12.2) and the Fortinsky Caregiver Self‐Efficacy Scale (52.9 to 64.8).

**Conclusion:**

PLWCI and care partners were integrally engaged in the co‐creation of a two‐component novel intervention to improve ED‐to‐community care transitions.

